# Joint QTL mapping and gene expression analysis identify positional candidate genes influencing pork quality traits

**DOI:** 10.1038/srep39830

**Published:** 2017-01-05

**Authors:** Rayner González-Prendes, Raquel Quintanilla, Angela Cánovas, Arianna Manunza, Tainã Figueiredo Cardoso, Jordi Jordana, José Luis Noguera, Ramona N. Pena, Marcel Amills

**Affiliations:** 1 Center for Research in Agricultural Genomics (CSIC-IRTA-UAB-UB), Campus Universitat Autònoma de Barcelona, Bellaterra 08193, Spain; 2 Animal Breeding and Genetics Program, Institut de Recerca i Tecnologia Agroalimentàries (IRTA), Torre Marimon, Caldes de Montbui 08140, Spain; 3 CAPES Foundation, Ministry of Education of Brazil, Brasilia D. F., Zip Code 70.040-020, Brazil; 4 Departament de Ciència Animal i dels Aliments, Universitat Autònoma de Barcelona, Bellaterra 08193, Spain; 5 Department of Animal Science, University of Lleida - Agrotecnio Center, Lleida 25198, Spain

## Abstract

Meat quality traits have an increasing importance in the pig industry because of their strong impact on consumer acceptance. Herewith, we have combined phenotypic and microarray expression data to map loci with potential effects on five meat quality traits recorded in the *longissimus dorsi* (LD) and *gluteus medius* (GM) muscles of 350 Duroc pigs, *i.e.* pH at 24 hours post-mortem (pH_24_), electric conductivity (CE) and muscle redness (a*), lightness (L*) and yellowness (b*). We have found significant genome-wide associations for CE of LD on SSC4 (~104 Mb), SSC5 (~15 Mb) and SSC13 (~137 Mb), while several additional regions were significantly associated with meat quality traits at the chromosome-wide level. There was a low positional concordance between the associations found for LD and GM traits, a feature that reflects the existence of differences in the genetic determinism of meat quality phenotypes in these two muscles. The performance of an eQTL search for SNPs mapping to the regions associated with meat quality traits demonstrated that the GM a* SSC3 and pH_24_ SSC17 QTL display positional concordance with cis-eQTL regulating the expression of several genes with a potential role on muscle metabolism.

The physicochemical properties of the porcine muscle and its post-mortem maturation determine the organoleptic properties of fresh meat and cured products and, consequently, their acceptance by consumers^1^. The genetic determinism of electrical conductivity, acidity and color, which have been often used as predictors of meat quality, has been explored by performing genome-wide association studies (GWAS) in F_2_ populations^2^,^3^,^4^ as well as in purebred pigs^5^,^6^. An important limitation of using F_2_ intercrosses in GWAS studies is that they are not representative of the purebred populations that constitute the selection nuclei of breeding companies. On the other hand, certain breeds, such as Large White, have been strongly introgressed with Asian alleles that do not segregate in other European porcine populations^7^.

In a previous study, we measured electrical conductivity at 24 hours (CE), pH at 24 hours (pH_24_) and color (lightness or L*, redness or a*, and yellowness or b*) in *gluteus medius* (GM) and *longissimus dorsi* (LD) samples from 350 Duroc pigs (Lipgen population)^8^. Performance of a genome scan with 105 microsatellites revealed that the QTL maps for these two muscles were quite different^8^. Indeed, the only QTL that remained significant at the genome-wide level were those associated with GM a*, on *Sus scrofa* chromosome 13 (SSC13, 84 cM), and GM b* (SSC15, 108 cM). Unfortunately, the confidence intervals of these QTL were quite large due to the poor resolution of the microsatellite-based analysis. Moreover, we may have missed many QTL due to the relatively large spacing between markers. In the current work, we aimed to circumvent these limitations by employing a GWAS approach to identify meat quality QTL in the Lipgen population mentioned above. Taking advantage that microarray measurements of gene expression in the GM muscle were available for 104 Lipgen pigs, we have performed an additional analysis where we have investigated the co-localization between GM QTL and expression QTL in *cis* (cis-eQTL).

## Materials and Methods

### Ethics approval

The manipulation of Duroc pigs followed Spanish national guidelines and it was approved by the Ethical Committee of Institut de Recerca i Tecnologia Agroalimentàries (IRTA).

### Measurement of phenotypic and expression data

Phenotypic records were collected in a commercial Duroc line of 350 barrows distributed in five half-sib families (Lipgen population). A detailed description of the management conditions of this commercial line has been previously reported^9^. Meat quality analyses were performed 24 h after slaughter at the IRTA-Centre of Food Technology by using 200 g samples of the LD and GM muscles. Electrical conductivity was estimated with a Pork Quality Meter (Intek GmbH) while pH_24_ was measured with a pH-meter equipment with a Xerolyte electrode (Crison). Meat L*, a* and b* color parameters were determined with a Minolta Chroma-Meter CR-200 (Konica Minolta) equipment (light source C and aperture 2). Microarray expression data of GM samples from 104 Duroc pigs were obtained in a previous study (data can be found in the Gene Expression Omnibus public repository, accession number: GSE19275) based on the use of GeneChip Porcine Genomic arrays (Affymetrix, Inc., Santa Clara, CA)^10^. A detailed description of the techniques and methods used to perform the RNA purification and microarray hybridization steps can be found in Canovas *et al*.^10^. Briefly, GM samples from 104 pigs were grinded in liquid nitrogen and homogenized with a mechanical rotor. Total RNA was purified with an acid phenol protocol^11^ and it was subsequently used as a template to synthesize double stranded cDNA with the One Cycle cDNA Synthesis Kit (Affymetrix, Inc.). cRNAs were purified with the GeneChip Sample Cleanup Module (Affymetrix, Inc.), fragmented and added to a hybridisation cocktail^10^. The GeneChip Porcine Genome Array was equilibrated to room temperature and prehybridised with 1× hybridisation buffer at 45 °C for 10 min^10^. The hybridisation cocktail was heated to 99 °C for 5 min in a heat block and cooled to 45 °C for 5 min. Subsequently, a hybridization step was carried out at 45 °C for 16 hours. GeneChips were washed and labeled with streptavidin phycoerythrin in a Fluidics Station 450 (Affymetrix, Inc) and they were scanned in an Agilent G3000 GeneArray Scanner (Agilent Technologies, Inc.). The “Affy” and “Sympleaffy” packages from the Bioconductor project^12^ were employed to establish a set of quality control metrics to assess the quality of RNA samples and the efficiencies of the labelling and hybridisation steps. Data pre-processing and normalization were carried out with the BRB-ArrayTools software version 3.7.1^13^. Genes displaying more than 20% of expression values over ±1.5 times the median expression of all arrays were retained for further analysis.

### Genome-wide association analysis for meat quality and expression data

Genotyping was performed with the Porcine SNP60 BeadChip (Illumina, San Diego, CA) which contains 62,163 single nucleotide polymorphisms (SNPs). Quality genotyping analyses were carried out with the GenomeStudio software (Illumina), as previously reported^14^. We removed SNPs (a) mapping to the X chromosome, (b) with a rate of missing genotypes higher than 5%, (c) that did not conform Hardy-Weinberg expectations (threshold set at a *P*-value ≤ 0.001), (d) that had a minor allele frequency below 0.05, (e) that had a GenCall score < 0.15, (f) that had a call rate < 95% or (g) that could not be mapped to the pig genome (*Sus scrofa* 10.2 assembly). After filtering the raw data, a GWAS was carried out with 36,710 SNPs. Single-SNP association analyses were performed with the Genome-wide Efficient Mixed-Model Association (GEMMA) software^15^ under an additive genetic model that included the genomic kinship matrix to account for relatedness. The statistical model assumed in this analysis was:


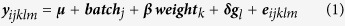


where ***y***_*ijklm*_ is the vector of phenotypic observations *i.e.* pH_24_, CE, L*, a* and b* measured at the GM and LD muscles of the *i*^*th*^ individual; ***μ*** is the population mean of each trait; ***batch***_*j*_ is a systematic effect of the *j*^*th*^ fattening batch, with 4 categories; ***β*** is the regression coefficient on the covariate *weight at slaughter (**weight***_*k*_); ***δ*** is the SNP allelic effect, estimated as a regression coefficient on the corresponding ***g***_*l*_ genotype (values −1, 0, 1) of the *l*^*th*^ SNP; and ***e***_*ijklm*_ is the residual effect. The statistical relevance of the systematic environmental sources of variation and the covariates included in the model were previously reported by Gallardo *et al*.^8^ and Casellas *et al*.^16^. Correction for multiple testing was implemented with a false discovery rate approach^17^.

Microarray data were available exclusively for GM muscle samples^10^. Following the strategy employed in the Genotype-Tissue Expression (GTEx) pilot analysis^18^, we primarily searched for cis-eQTL because they are expected to have larger effects than their trans-counterparts. We used two different strategies: **Analysis 1**, we retrieved 12 genes localized within GM QTL regions and we looked for cis-eQTL that might regulate their expression and **Analysis 2**, we made a search for cis-eQTL at a whole genome scale and we analyzed if there was a positional concordance between GWAS signals and cis-eQTL identified in this way. This second strategy made possible to identify cis-eQTL that might be located in the vicinity of GWAS signals. Genes corresponding to each probe included in the GeneChip Porcine Genomic array (Affymetrix, Inc., Santa Clara, CA) were identified in the BioMart database^19^. The statistical model assumed in this analysis was:


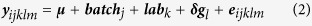


where ***y***_*ijklm*_ is the vector that defines the expression of each gene in the GM muscle of the *i*^*th*^ individual; **μ** is the mean expression of each gene in the population; ***batch***_*j*_and ***lab***_*k*_ are the systematic effects *i.e.* ***batch***_*j*_ of fattening (with 4 categories) and ***lab***_*k*_ (microarray data were generated in two different laboratories); ***δ*** is the SNP allelic effect estimated as a regression coefficient on the corresponding ***g***_*l*_ genotype (values −1, 0, 1) of the *l*^*th*^ SNP; and ***e***_*ijklm*_ is the residual effect. Correction for multiple testing was implemented with a false discovery rate approach^17^. The threshold of significance in **Analysis 1** took into consideration the number of SNPs contained within 2 Mb windows around each one of the 12 genes under consideration, while in **Analysis 2** such threshold was established by taking into account the 36,710 SNPs typed in the Duroc population.

## Results and Discussion

### The SNPs arrayed in the Porcine SNP60 BeadChip explain a limited amount of the phenotypic variance of meat quality traits

By using the GEMMA software, we have estimated the proportion of phenotypic variance explained by the 36,710 SNPs (h^2^_SNP_) genotyped with the Porcine SNP60 BeadChip (Table 1). In general, estimates of h^2^_SNP_ ranged from low to moderate and differed between muscles. Discrepancies in the genealogic heritability (h^2^) estimates of meat quality traits recorded in different skeletal muscle samples were previously reported by Larzul *et al*.^20^. In this way, these authors found h^2^ of 0.03 and 0.23 for L* measured in the *gluteus profundus* and *longissimus* muscles, respectively. Similarly, the h^2^ values of pH_24_ measured in 4 different muscles oscillated between 0.17 (*longissimus*) and 0.39 (*biceps femoris*)^20^. When Gallardo *et al*.^8^ performed a QTL scan for meat quality traits in the Lipgen population, they also found that QTL maps differed markedly amongst traits recorded in the GM and LD muscles. As a whole, these results suggest that there are muscle-specific factors that modulate the genetic determinism of meat quality traits. Indeed, Quintanilla *et al*.^21^ identified remarkable differences in the gene expression patterns of the LD and GM muscles, a feature that was especially prominent for genes involved in muscle tissue development, cell proliferation and migration and muscle contraction.

Several h^2^_SNP_ values obtained by us were comparable to genealogic heritabilities estimated for porcine meat quality traits in previous studies. For instance Gjerlaug-Enger *et al*.^22^ reported heritabilities for a* of 0.43 and 0.46 in Duroc and Landrace pigs, respectively. Similarly, Van Wijk *et al*.^23^ and Gjerlaug-Enger *et al*.^22^ described heritabilities of 0.11 (crossbred pigs) and from 0.12 (Landrace) to 0.27 (Duroc) for pH_24_. More unexpected were the null h^2^_SNP_ values obtained in the current work for traits such as b* (in GM) and L* (in both muscles). We attribute these null heritabilities to our inability to detect genetic variants that may have small effects or that segregate at very low frequencies^24^.

Environmental variables may also obscure the contribution of genetic factors. Indeed, meat quality traits can be affected by poor on-farm handling, mixing of unfamiliar animals and high pig density and long travel distance during transportation^25^. Such events may increase the stress of the swine brought to the abattoir and, consequently, they may have negative consequences on meat quality^25^. At the abattoir, extended lairage time can increase the incidence of dark, firm and dry (DFD) meat, while a short lairage time has been associated with an increased proportion of pale, soft and exudative (PSE) meat^25^. Electrical stunning induces a more rapid pH fall early post mortem and an inferior water-holding capacity than CO_2_ stunning, while an accelerated chilling may have negative consequences on meat tenderness and water-holding capacity^25^. In summary, all these factors, and others that are not mentioned, can have a strong impact on the post-mortem pH, electrical conductivity and color of pig meat and “dilute” the contribution of polygenes^25^.

### Genome-wide and chromosome-wide associations with meat quality traits in Duroc pigs

At the genome-wide level, we found significant associations between CE of LD and three genomic regions on SSC4, SSC5 and SSC13 (Table 2). The SSC4, 104 megabase (Mb) region, lies close to a previously reported QTL for CE identified by Cepica *et al*.^26^. We also found positional concordance between the SSC13 (137.0 Mb) region associated with LD CE and a *semimembranosus* CE QTL reported by Evans *et al*.^27^. At the chromosome-wide level, a coincidence was detected between a a* QTL on SSC3 (50–57 Mb, Table 3) and a QTL for the same trait reported by Li *et al*.^28^ on SSC3 (55 Mb). Overall, our results confirm the existence of differences in the genetic determinism of meat quality traits recorded in the GM and LD muscles. The only exception was a region on SSC5 that significantly affected CE in both LD and GM muscles (Table 3). When we compared these data with the set of QTL previously reported by Gallardo *et al*.^8^ in the same Lipgen population we found one coincidence *i.e.* the GWAS signal identified on SSC4 (132 Mb) for CE in LD overlapped the confidence interval of a LD CE QTL (S0097 marker, ~133 Mb) detected by these authors^8^.

In general the positional coincidence between GWAS signals detected by us and those reported in previous studies was weak, indicating that the majority of associations reported in the current work are new. For instance, when we compared our a*, b* and pH_24_ data with those described in six additional GWAS studies^4^,^6^,^29^,^30^,^31^,^32^ we only found one positional coincidence between the SSC10 (70.6 Mb) genomic region associated with LD a* in the Lipgen population (Table 3) and the SSC10 (72.8 Mb) region identified by Ma *et al*.^4^ as associated with the same trait in the *semimembranosus* muscle of White Duroc × Erhualian F_2_ pigs.

The level of coincidence of trait-associated regions between these six GWAS for a*, b* and pH_24_ traits was also quite low. Only about 20% of the regions identified as significantly associated with any of these phenotypes were shared between two studies or more, indicating that the majority of associations are population-specific. These shared regions were: (a*) SSC4 (80–85 Mb)^6^,^30^, SSC6 (17–22 Mb)^4^,^30^, SSC7 (31–32 Mb)^4^,^31^, SSC12 (58–63 Mb)^30^,^31^, SSC15 (133–136 Mb)^30^,^31^,^32^; (b*) SSC15 (129–133 Mb)^30^,^32^; and (pH_24_), SSC3 (15–19 Mb)^30^,^31^, SSC15 (133–136 Mb)^29^,^32^. This latter region on SSC15 (133–136 Mb) appeared to be associated with a*, b*, pH_24_, shear force and cook loss in many independent studies^29^,^30^,^31^,^32^ but not in ours. Interestingly, this SSC15 region contains the protein kinase AMP-activated non-catalytic subunit gamma 3 (*PRKAG3*) gene, whose polymorphism has causal effects on muscle glycogen depletion, a parameter that can have a strong influence on meat quality traits^33^.

Besides technical and methodological reasons, a probable cause for the lack of positional concordance between GWAS studies would be genetic heterogeneity^34^. Indeed, Yang *et al*.^34^ performed a GWAS for blood lipid traits in 2,400 Laiwu, Erhualian and Duroc × (Landrace × Yorkshire) pigs and they identified a total of 22 QTL. Notably, only six regions were identified in more than one population, and 16 were detected in a single population.

### Positional concordance between cis-eQTL for genes expressed in the GM muscle and QTL for GM traits

In general, eQTL are highly enriched in variants with causal effects on phenotypic variation and they can provide valuable information about candidate genes to be further investigated. Integrative analyses of QTL and eQTL data have been performed in pigs, making possible to combine the power of recombination with expression studies in order to identify promising candidate genes^35^. For instance, multiple associations between SNPs mapping to porcine chromosomes 4 and 6 and meat quality traits have been detected^30^. Through an eQTL approach, it was possible to identify several genes on SSC4 (*ZNF704, IMPA1* and *OXSR1*) and SSC6 (*IH1D1, SIGLEC10, TBCB, LOC100518735, KIF1B, LOC100514845*) whose variation is concomitantly associated with gene expression and phenotype data^30^. Similarly, Ma *et al*.^36^ used a genetical genomics approach to demonstrate that a splice mutation in the *PHKG1* gene is the causal mutation for a glycolytic potential QTL mapping to SSC3.

**Figure 1:**
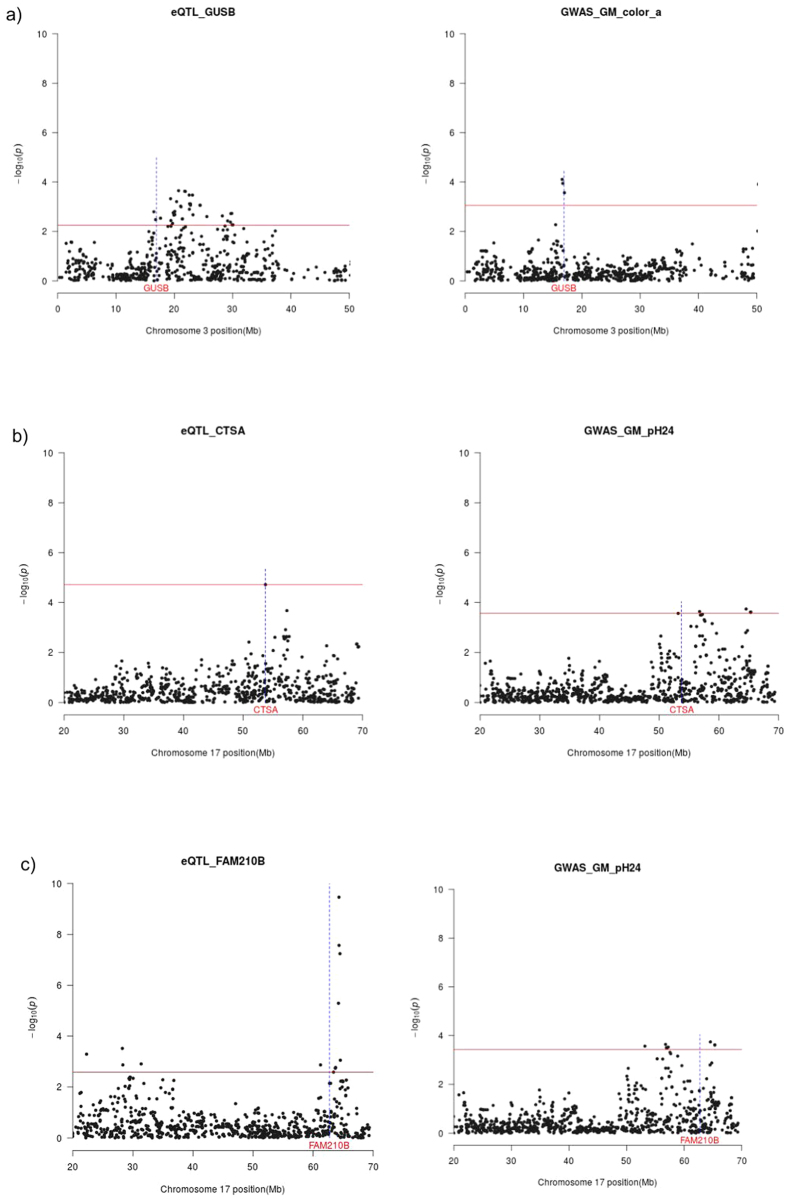
Cis-eQTL (left panel) for the *GUSB* (1**a**), *CTSA* (1**b**) and *FAM210B* (1**c**) genes which map to QTL regions associated with meat quality traits recorded in the *gluteus medius* muscle (right panel). The *x*-axis represents chromosome length (Mb), and the *y*-axis shows the −log10 (*P*-value) of the associations found. The horizontal line indicates the threshold of significance (*q*-value ≤ 0.05). The vertical line depicts the genomic location of the *GUSB, CTSA* and *FAM210B* genes.

We have used this integrative strategy to identify potential candidate genes for meat quality traits in a dataset of 12 loci that mapped to GM QTL regions (**Analysis 1**). In doing so, we have detected 3 cis-eQTLs (Table 4) that co-localize with three chromosome-wide QTLs. One of them maps to SSC3 (16.6–17.06 Mb) and displays associations with a* (Fig. 1a); while the other two are located on SSC17 (53.1–57.2; 64.5–65.3) and show significant associations with GM pH_24_ (Fig. 1b and c). Interestingly, two of the three cis-regulated genes encode lysosomal enzymes, *i.e.* cathepsin A (*CTSA*) and glucuronidase β (*GUSB*), that might be released during the post-mortem maturation of meat^37^,^38^. Cathepsin A is a lysosomal serine protease that can also protect galactosidase β from intralysosomal proteolysis^38^, while glucuronidase β is mainly involved in the degradation of glycosaminoglycans^39^. Interestingly, there are evidences that galactosidase β and glucuronidase β might affect the degradation of the collagen mucopolysaccharide, thus having a potential impact on meat ultrastructural properties^40^.

**Figure 2:**
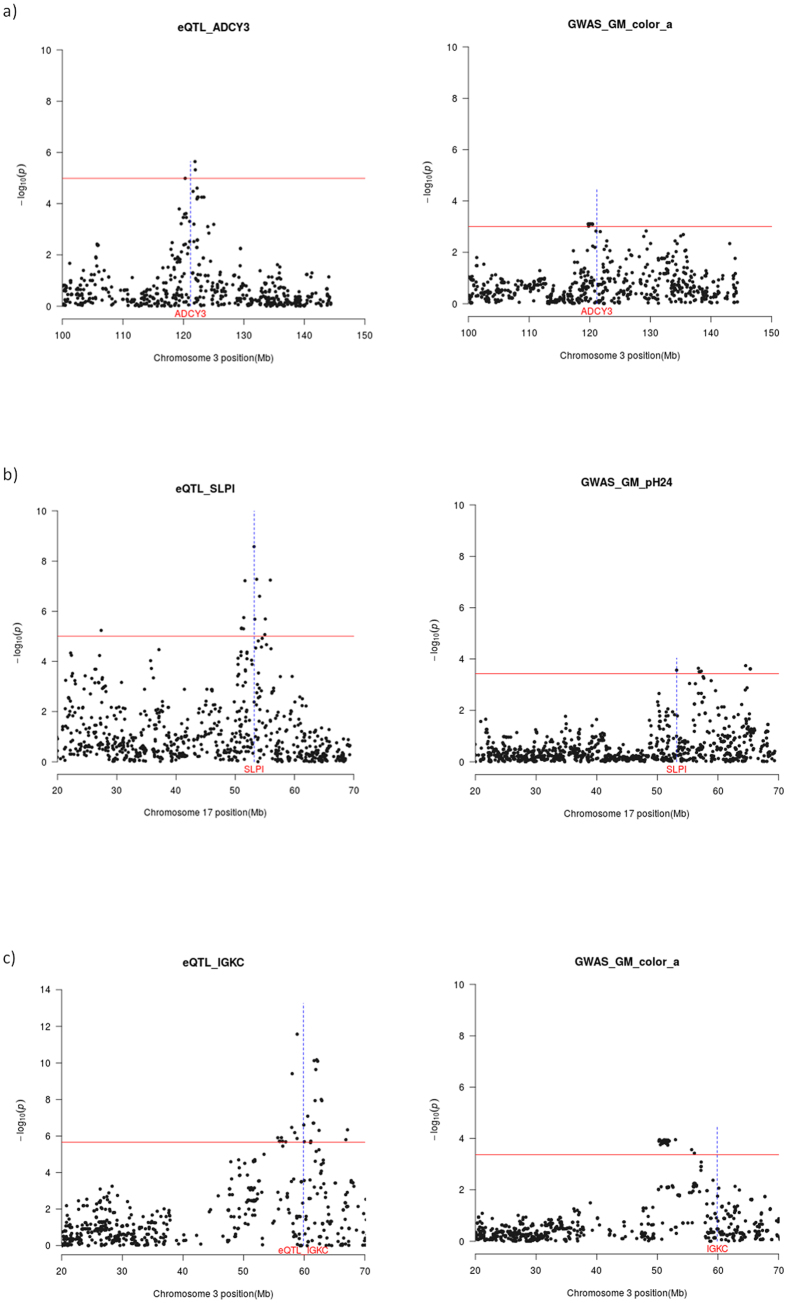
Co-localization of cis-eQTL (left panel) for the *ADCY3* (2**a**), *SLP1* (2**b**) and *IGKC* (2**c**) genes and QTL for meat quality traits recorded in the *gluteus medius* muscle (right panel). The *x*-axis represents chromosome length (Mb), and the *y*-axis shows the –log10 (*P*-value) of the associations found. The horizontal line indicates the threshold of significance (*q*-value ≤ 0.05). The vertical line depicts the genomic location of the *ADCY3, SLP1 and IGKC* genes.

In **Analysis 2**, we have identified three additional cis-eQTL that map near to the SSC3 QTL for a* and the SSC17 QTL for pH_24_ (Table 5). The *ADCY3* locus, that co-localizes with the SSC3 QTL for GM a* (Fig. 2a), encodes an adenylate cyclase catalysing the conversion of ATP into cyclic adenosine-3′,5′-monophosphate (cAMP), a secondary messenger that can have broad effects on muscle metabolism^41^. Indeed, AMPc is an activator of the cAMP-dependent protein kinase, a molecule involved in the phosphorylation of enzymes that promote the conversion of glycogen into glucose^41^. Noteworthy, the amount of glycogen stored in the muscle determines the post-mortem production of lactic acid, a molecule that has strong effects on meat color. Another eQTL of interest is the one influencing the mRNA levels of the secretory leukocyte peptidase inhibitor (*SLPI*) gene. This cis-eQTL co-localizes with the SSC17 QTL for GM pH_24_ (Fig. 2b). The *SLP1* gene encodes a serine-protease that inhibits protein-degrading enzymes with strong effects on meat tenderization *i.e*. when the skeletal muscle is being degraded and transformed into meat, SLPI attenuates muscle proteolysis by binding to proteases and rendering them inactive^42^. Finally, the co-localization of the *IGKC* cis-eQTL and the SSC3 QTL for a* (Fig. 2c) does not have an obvious biological interpretation because this gene is mainly related with humoral immunity.

## Conclusions

We have detected genome-wide and chromosome-wide significant QTL for meat quality traits recorded in a Duroc commercial line with a population size that was moderate but comparable to the ones used in other porcine GWAS^43^,^44^,^45^. The limited positional concordance between the set of QTL detected by us and those reported by other authors in purebred populations suggests the existence of a significant amount of genetic heterogeneity for meat quality traits in porcine breeds. We have found remarkable differences between the QTL maps for the LD and GM muscles, suggesting that meat quality is determined to a great extent by genetic factors that are muscle-specific. Finally, we have observed a number of cis-eQTL that co-localize with meat quality QTL regions. Several of these cis-eQTL regulate the expression of genes which may play important roles in muscle physiology and post-mortem meat maturation. Sequencing of the regulatory regions of these loci might be useful to uncover the identity of the causal mutations explaining the existence of these QTLs.

## Additional Information

**How to cite this article**: González-Prendes, R. *et al*. Joint QTL mapping and gene expression analysis identify positional candidate genes influencing pork quality traits. *Sci. Rep.* **7**, 39830; doi: 10.1038/srep39830 (2017).

**Publisher's note:** Springer Nature remains neutral with regard to jurisdictional claims in published maps and institutional affiliations.

## Figures and Tables

**Table 1:** Proportion of phenotypic variance of meat quality traits recorded in the *longissimus dorsi* (LD) and *gluteus medius* (GM) muscles of Duroc pigs explained by SNP markers (h^2^_SNP_) and its standard error (SE).

Phenotype	h^2^_SNP_ ± SE	
	LD muscle	GM muscle
Electric conductivity (CE)	0.20 ± 0.07	0.11 ± 0.08
pH at 24 hours (pH_24_)	0.17 ± 0.10	0.12 ± 0.09
Minolta redness (a*)	0.41 ± 0.11	0.45 ± 0.11
Minolta yellowness (b*)	0.29 ± 0.12	0.00 ± 0.14
Minolta lightness (L*)	0.00 ± 0.25	0.00 ± 0.05

**Table 2:** Genomic regions significantly associated at the genome-wide level with meat quality traits in Duroc pigs.

Trait	SSC	N	SNP	Location (Mb)	*P*-value	*q*-value	δ ± SE	A1	MAF
LD CE	4	4	H3GA0013593	104.2–104.8	6.19E-06	0.04	0.28 ± 0.06	A	0.39
	5	1	ASGA0024711	15.4	2.46E-06	0.04	−0.32 ± 0.07	G	0.18
	13	1	ALGA0027007	137.0	7.34E-06	0.04	0.27 ± 0.06	A	0.39

**LD:** *longissimus dorsi* muscle, **CE:** Electrical conductivity at 24 hours post-mortem, **N**: Number of SNPs significantly associated with the trait under study, **SSC**: porcine chromosome, **SNP**: SNP displaying the most significant association with the trait under study, **Location (Mb)**: region containing SNPs significantly associated with the trait under study, ***P*****-value**: nominal P-value, ***q*****-value**: q-value calculated with a false discovery rate approach, ***δ***: allelic effect and its standard error (**SE**), **A1**: minority allele, **MAF**: frequency of the minority allele.

**Table 3:** Genomic regions associated at the chromosome-wide level with meat quality traits in Duroc pigs.

Trait	SSC	N	SNP	Location (Mb)	*P-*value	*q-*value	δ ± SE	A1	MAF
LD CE	4	9	ALGA0026686	93.5–98.8	1.54E-05	0.01	−0.28 ± 0.06	G	0.50
		32	H3GA0013593	104.2–107.1	6.19E-06	0.01	0.28 ± 0.06	A	0.39
		1	ALGA0028809	131.0	2.04E-04	0.02	−0.26 ± 0.07	A	0.17
	5	11	ASGA0024711	14.4–16.1	2.46E-06	0.004	−0.32 ± 0.07	G	0.18
GM CE	5	5	ASGA0024564	13.0–14.7	3.15E-05	0.03	−0.37 ± 0.09	A	0.39
LD pH_24_	16	3	MARC0086782	6.0–6.4	5.27E-04	0.05	0.08 ± 0.02	G	0.09
		2	ALGA0089269	17.3–18.5	5.09E-04	0.05	−0.06 ± 0.02	G	0.19
		10	ASGA0091353	20.9–29.5	4.01E-04	0.05	0.05 ± 0.02	G	0.41
GM pH_24_	17	2	MARC0038923	14.2–16.4	9.11E-05	0.04	−0.06 ± 0.02	A	0.48
		5	MARC0101162	53.1–57.2	2.70E-04	0.04	0.07 ± 0.02	G	0.29
		3	H3GA0049744	64.5–65.3	1.81E-04	0.04	−0.06 ± 0.02	G	0.38
LD a*	10	1	ALGA0113811	70.6	2.99E-05	0.04	0.46 ± 0.11	A	0.36
GM a*	3	3	H3GA0009494	16.6–17.0	7.85E-05	0.01	0.70 ± 0.17	A	0.16
		27	H3GA0009489	50.2–57.2	1.27E-04	0.01	0.65 ± 0.17	A	0.18
		4	ALGA0021059	119.7–119.9	7.85E-04	0.04	0.48 ± 0.14	A	0.24
		4	ALGA0021078	120.0–120.4	7.85E-04	0.04	0.48 ± 0.14	A	0.24
GM L*	16	1	MARC0073433	3.5	3.45E-05	0.04	1.23 ± 0.29	C	0.24

**GM**: *gluteus medius* muscle, **LD:** *longissimus dorsi* muscle, **CE**: Electrical conductivity at 24 hours post-mortem, **pH**_**24**_: pH at 24 hours post-mortem; a^*^: Minolta redness; L^*^: Minolta lightness, **N**: Number of SNPs significantly associated with the trait under study, **SSC**: porcine chromosome, **SNP**: SNP displaying the most significant association with the trait under study, **Location (Mb)**: region containing SNPs significantly associated with the trait under study, ***P*****-value**: nominal P-value, ***q*****-value**: q-value calculated with a false discovery rate approach, ***δ***: allelic effect and its standard error (**SE**), **A1**: minority allele, **MAF**: frequency of the minority allele.

**Table 4:** List of significant cis-eQTLs mapping within QTL regions for *gluteus medius* meat quality traits.

QTLs			Genes			Cis-eQTLs									
Trait	SSC	Location (Mb)	Names	SSC	Location (Mb)	SSC	N	SNPs	Location (Mb)	*P-*value	*q-*value	*B*	δ ± SE	A1	MAF
GM a*	3	16.6–17.0	*GUSB*	3	16.9	3	3	ALGA0104024	16.4–17.6	1.60E-03	0.02	0.04	0.28 ± 0.09	A	0.46
GM pH_24_	17	53.1–57.2	*CTSA*	17	53.7	17	1	ALGA0095491	53.7	1.91E-05	6.11E-04	6.11E-04	−0.37 ± 0.08	G	0.25
		64.5–65.3	*FAM210B*		64.0		16	ALGA0096195	64.1–65.7	4.53E-11	1.99E-09	1.99E-09	−0.53 ± 0.07	G	0.22

a^*^: Minolta redness, **pH_24_**: pH at 24 hours post-mortem**, N:** number of significant SNPs, **SNP**: marker displaying the most significant association with the trait under study, **Location (Mb)**: region containing SNPs significantly associated with the trait under study, ***P*****-value:** nominal P-value, ***q*****-value:** q-value calculated with a false discovery rate approach, ***B***: P-value corrected for multiple testing with the Bonferroni method, ***δ***: allelic effect and its standard error (**SE**), **A1:** minority allele, **MAF:** frequency of the minority allele.

**Table 5:** List of significant cis-eQTLs mapping close to QTL regions for *gluteus medius* meat quality traits.

QTLs			Genes			Cis-eQTLs									
Traits	SSC	Location (Mb)	Names	SSC	Location (Mb)	SSC	N	SNPs	Location (Mb)	*P-*value	*q-*value	*B*	δ ± SE	A1	MAF
GM a*	3	50.2–57.2	*IGKC*	3	59.8	3	20	ALGA0019294	58.0–61.9	7.54E-11	4.60E-07	2.15E-06	−1.6 ± 0.22	A	0.19
		120.0–120.4	*ADCY3*		121.1–121.2		3	ALGA0103469	120.0–121.9	2.28E-06	0.05	0.06	−0.83 ± 0.17	A	0.08
GM pH_24_	17	53.1–57.2	*SLPI*	17	53.1	17	16	ALGA0095584	52.3–55.9	6.00E-08	3.48E-04	1.64E-03	2.30 ± 0.40	A	0.13

a^*^: Minolta redness, **pH**_**24**_: pH at 24 hours post-mortem, **N:** number of significant SNPs, **SNPs**: marker displaying the most significant association with the trait under study, **Location (Mb)**: region containing SNPs significantly associated with the trait under study, ***P*****-value:** nominal P-value, ***q*****-value:** q-value calculated with a false discovery rate approach, ***B***: P-value corrected for multiple testing with the Bonferroni method, ***δ***: allelic effect and its standard error (**SE**), **A1:** minority allele, **MAF:** frequency of the minority allele.

## References

[b1] Ciobanu D. C., Lonergan S. M. & Huff-Lonergan E. J. Genetics of meat and carcass traits In The Genetics of the Pig. 2nd edn. (ed. Rothschild M. F. & Ruvinsky A.) 356 (CABI, 2011).

[b2] Luo W. . Genome-wide association analysis of meat quality traits in a porcine Large White × Minzhu intercross population. Int J Biol Sci. 8, 580–595 (2012). 22532790 10.7150/ijbs.3614 PMC3334672

[b3] Nonneman D. . Genome-wide association of meat quality traits and tenderness in swine. J. Anim. Sci. 91, 4043–4050 (2013). 23942702 10.2527/jas.2013-6255

[b4] Ma J. . Genome-wide association study of meat quality traits in a White Duroc × Erhualian F_2_ intercross and Chinese Sutai pigs. PloS One 8, e64047 (2013). 23724019 10.1371/journal.pone.0064047 PMC3665833

[b5] Becker D., Wimmers K., Luther H., Hofer A. & Leeb T. A Genome-wide association study to detect QTL for commercially important traits in Swiss Large White boars. PloS One 8, e5595 (2013). 10.1371/journal.pone.0055951 PMC3564845 23393604

[b6] Sanchez M. P. . Genome-wide association study of production traits in a commercial population of Large White pigs: evidence of haplotypes affecting meat quality. Genet Sel Evol. 46, 12 (2014). 24528607 10.1186/1297-9686-46-12 PMC3975960

[b7] Fang M. & Andersson L. Mitochondrial diversity in European and Chinese pigs is consistent with population expansions that occurred prior to domestication. Proc. Biol. Sci. 273, 1803–1810 (2006). 16790414 10.1098/rspb.2006.3514 PMC1634785

[b8] Gallardo D. . Quantitative trait loci analysis of a Duroc commercial population highlights differences in the genetic determination of meat quality traits at two different muscles. Anim. Genet. 43, 800–804 (2012). 22497576 10.1111/j.1365-2052.2012.02333.x

[b9] Gallardo D. . Polymorphism of the pig acetyl-coenzyme A carboxylase α gene is associated with fatty acid composition in a Duroc commercial line. Anim. Genet. 40, 410–417 (2009). 19392830 10.1111/j.1365-2052.2009.01854.x

[b10] Canovas A., Quintanilla R., Amills M. & Pena R. N. Muscle transcriptomic profiles in pigs with divergent phenotypes for fatness traits. BMC Genomics 11, 372 (2010). 20540717 10.1186/1471-2164-11-372 PMC2894043

[b11] Chomczynski P. & Sacchi N. Single-step method of RNA isolation by acid guanidinium thiocyanate-phenol-chloroform extraction. Anal. Biochem. 162, 156–159 (1987). 2440339 10.1006/abio.1987.9999

[b12] Gentleman R. C. . Bioconductor: open software development for computational biology and bioinformatics. Genome Biol. 5, R80 (2004). 15461798 10.1186/gb-2004-5-10-r80 PMC545600

[b13] Xu X., Zhao Y. & Simon R. Gene set expression comparison kit for BRB-Array tools. Bioinformatics 24, 137–139 (2008). 18006549 10.1093/bioinformatics/btm541

[b14] Manunza A. . A genome-wide association analysis for porcine serum lipid traits reveals the existence of age-specific genetic determinants. BMC Genomics 15, 758 (2014). 25189197 10.1186/1471-2164-15-758 PMC4164741

[b15] Zhou X. & Stephens M. Genome-wide efficient mixed-model analysis for association studies. Nat. Genet. 44, 821–824 (2012). 22706312 10.1038/ng.2310 PMC3386377

[b16] Casellas J. . Bayes factor analyses of heritability for serum and muscle lipid traits in Duroc pigs. J. Anim. Sci. 88, 2246–2254 (2010). 20418459 10.2527/jas.2009-2205

[b17] Benjamini Y. & Hochberg Y. Controlling the false discovery rate: a practical and powerful approach to multiple testing. J. R. Stat. Soc. Ser. B 57, 289–300 (1995).

[b18] GTEx Consortium. Human genomics. The Genotype-Tissue Expression (GTEx) pilot analysis: multitissue gene regulation in humans. Science 348, 648–660 (2015). 25954001 10.1126/science.1262110 PMC4547484

[b19] Smedley D. . The BioMart community portal: an innovative alternative to large centralized data repositories. Nucleic Acids Res. 43, W589–598 (2015). 25897122 10.1093/nar/gkv350 PMC4489294

[b20] Larzul C. . Selection for reduced muscle glycolytic potential in Large White pigs. II. Correlated responses in meat quality and muscle compositional traits. Genet. Sel. Evol. 31, 61–76 (1999).

[b21] Quintanilla R., Pena R. N., Cánovas A. & Amills M. Differential gene expression profile between two porcine skeletal muscles: *longissimus dorsi* and *gluteus medius*. In Book of abstracts of the 32th International Conference on Animal Genetics (2010).

[b22] Gjerlaug-Enger E., Aass L., Ødegard J. & Vangen O. Genetic parameters of meat quality traits in two pig breeds measured by rapid methods. Animal 4, 1832–1843 (2010). 22445144 10.1017/S175173111000114X

[b23] Van Wijk H. J. . Genetic parameters for carcass composition and pork quality estimated in a commercial production chain. J. Anim. Sci. 83, 324–333 (2005). 15644503 10.2527/2005.832324x

[b24] Zuk O., Hechter E., Sunyaev S. & Lander E. The mystery of missing heritability: Genetic interactions create phantom heritability. Proc. Natl. Acad. Sci. USA 109, 1193–1198 (2012). 22223662 10.1073/pnas.1119675109 PMC3268279

[b25] Rosenvold K. & Andersen H. J. Factors of significance for pork quality-a review. Meat Sci. 64, 219–237 (2003). 22063008 10.1016/S0309-1740(02)00186-9

[b26] Cepica S. . Linkage and QTL mapping for sus scrofa chromosome 4. J. Anim. Breed. Genet. 120, 28–37 (2003).

[b27] Evans G. J. . Identification of quantitative trait loci for production traits in commercial pig populations. Genetics 164, 621–627 (2003). 12807782 10.1093/genetics/164.2.621 PMC1462582

[b28] Li H. D. . Quantitative trait loci analysis of swine meat quality traits. J. Anim. Sci. 88, 2904–2912 (2010). 20495113 10.2527/jas.2009-2590

[b29] Bernal Rubio Y. L. . Implementing meta-analysis from genome-wide association studies for pork quality traits. J. Anim. Sci. 93, 5607–5617 (2015). 26641170 10.2527/jas.2015-9502

[b30] Ponsuksili S., Murani E., Trakooljul N., Schwerin M. & Wimmers K. Discovery of candidate genes for muscle traits based on GWAS supported by eQTL-analysis. Int. J. Biol. Sci. 10, 327–337 (2014). 24643240 10.7150/ijbs.8134 PMC3957088

[b31] Liu X. . Genome-wide association analyses for meat quality traits in Chinese Erhualian pigs and a Western Duroc × (Landrace × Yorkshire) commercial population. Genet. Sel. Evol. 47, 44 (2015). 25962760 10.1186/s12711-015-0120-x PMC4427942

[b32] Zhang C. . Genome-wide association studies (GWAS) identify a QTL close to *PRKAG3* affecting meat pH and colour in crossbred commercial pigs. BMC Genetics 16, 33 (2015). 25887635 10.1186/s12863-015-0192-1 PMC4393631

[b33] Milan D. . A mutation in *PRKAG3* associated with excess glycogen content in pig skeletal muscle. Science 288, 1248–1251 (2000). 10818001 10.1126/science.288.5469.1248

[b34] Yang H. . Genome-wide association analysis for blood lipid traits measured in three pig populations reveals a substantial level of genetic heterogeneity. PloS One 10, e0131667 (2015). 26121138 10.1371/journal.pone.0131667 PMC4488070

[b35] Ernst C. W. & Steibel J. P. Molecular advances in QTL discovery and application in pig breeding. Trends Genet. 29, 215–224 (2013). 23498076 10.1016/j.tig.2013.02.002

[b36] Ma J. . A Splice mutation in the *PHKG1* gene causes high glycogen content and low meat quality in pig skeletal muscle. PLoS Genet. 10, e1004710 (2014). 25340394 10.1371/journal.pgen.1004710 PMC4207639

[b37] Dutson T. R. & Lawrie R. A. Release of lysosomal enzymes during post mortem conditioning and their relationship to tenderness. Int. J. Food Sci. Tech. 9, 43–50 (2007).

[b38] Hiraiwa M. Cathepsin A/protective protein: an unusual lysosomal multifunctional protein. Cell. Mol. Life Sci. 56, 894–907 (1999). 11212324 10.1007/s000180050482 PMC11146757

[b39] Naz H. . Human β-glucuronidase: structure, function, and application in enzyme replacement therapy. Rejuvenation Res. 16, 352–363 (2013). 23777470 10.1089/rej.2013.1407

[b40] Chang H. J., Xu X. L., Zhou G. H., Li C. B. & Huang M. Effects of characteristics changes of collagen on meat physicochemical properties of beef semitendinosus muscle during ultrasonic processing. Food Bioprocess. Technol. 5, 285–297 (2009).

[b41] Hong S. *et al. et al*. Upregulation of adenylate cyclase 3 (*ADCY3*) increases the tumorigenic potential of cells by activating the CREB pathway. Oncotarget 4, 1791–1803 (2013). 24113161 10.18632/oncotarget.1324 PMC3858564

[b42] Urso L. . Alterations in mRNA expression and protein products following spinal cord injury in humans. J. Physiol. 579, 877–892 (2007). 17218363 10.1113/jphysiol.2006.118042 PMC2151363

[b43] Ramayo-Caldas Y. . Genome-wide association study for intramuscular fatty acid composition in an Iberian × Landrace cross. J. Anim. Sci. 90, 2883–2893 (2012). 22785162 10.2527/jas.2011-4900

[b44] Fontanesi L. . A genome wide association study for backfat thickness in Italian Large White pigs highlights new regions affecting fat deposition including neuronal genes. BMC Genomics 13, 583 (2012). 23153328 10.1186/1471-2164-13-583 PMC3499287

[b45] Becker D., Wimmers K., Luther H., Hofer A. & Leeb T. A genome-wide association study to detect QTL for commercially important traits in Swiss Large White boars. PLoS One 8, e55951 (2013). 23393604 10.1371/journal.pone.0055951 PMC3564845

